# Relationship between Family Racial/Ethnic Backgrounds, Parenting Practices and Styles, and Adolescent Eating Behaviors

**DOI:** 10.3390/ijerph19127388

**Published:** 2022-06-16

**Authors:** Lillie Monroe-Lord, Alex Anderson, Blake L. Jones, Rickelle Richards, Marla Reicks, Carolyn Gunther, Jinan Banna, Glade L. Topham, Karina R. Lora, Siew Sun Wong, Miriam Ballejos, Laura Hopkins, Azam Ardakani

**Affiliations:** 1 Center for Nutrition, Diet and Health, University of the District of Columbia, Washington, DC 20008, USA; 2 Department of Nutritional Sciences, University of Georgia, Athens, GA 30602, USA; fianko@uga.edu; 3 Department of Psychology, Brigham Young University, Provo, UT 84602, USA; blake.jones@byu.edu; 4 Department of Nutrition, Dietetics & Food Science, Brigham Young University, Provo, UT 84602, USA; rickelle_richards@byu.edu; 5 Department of Food Science and Nutrition, University of Minnesota, St. Paul, MN 55108, USA; mreicks@umn.edu; 6 Human Nutrition Program, Department of Human Sciences, Ohio State University, Columbus, OH 43210, USA; gunther.22@osu.edu; 7 Department of Human Nutrition, Food and Animal Sciences, University of Hawaii-Manoa, Honolulu, HI 96822, USA; jcbanna@hawaii.edu; 8 Department of Applied Human Sciences, Kansas State University, Manhattan, KS 66506, USA; gtopham@ksu.edu; 9 Department of Exercise and Nutrition Sciences, The George Washington University, Washington, DC 20052, USA; klora@email.gwu.edu; 10 School of Biological and Population Health Sciences, Nutrition, Oregon State University, Corvallis, OR 97331, USA; siewsun.wong@oregonstate.edu; 11 Nutrition & Exercise Physiology, Washington State University, 412 E. Spokane Falls Blvd, Spokane, WA 99202, USA; miriam.s.ballejos.civ@mail.mil; 12 Department of Public Health and Prevention Sciences, Baldwin Wallace University, 275 Eastland Rd., Berea, OH 44017, USA; lhopkins@bw.edu; 13 Department of Nutritional Sciences, Howard University, Washington, DC 20059, USA; azam.ardakani@bison.howard.edu

**Keywords:** parenting practices, adolescent eating behavior, adolescent–parent dyads, race/ethnicity, unhealthy snacks, fruits and vegetables, dairy

## Abstract

Obesity is more prevalent among racial minority children in the United States, as compared to White children. Parenting practices can impact the development of children’s eating behaviors and habits. In this study, we investigated the relationships among racial/ethnic backgrounds, parenting practices and styles, and eating behaviors in adolescents. Fifty-one parent–adolescent dyads were interviewed to characterize parenting practices and styles, as well as the consumption of dairy, fruits and vegetables, and unhealthy snacks. Height and weight were measured to calculate parent BMI and adolescent BMI-for-age percentiles. Three parenting practice categories—modeling, authoritative, and authoritarian—were found to be related to race/ethnicity. A higher score in authoritarian parenting practices was related to higher BMI percentiles among African American adolescents, whereas a higher score in monitoring practices was related to lower BMI percentiles among non-Hispanic White adolescents. Modeling, reasoning, and monitoring led to higher consumption of fruits and vegetables among adolescents; however, the consumption of unhealthy snacks was higher with rule-setting and lower with reasoning and authoritative practices. Finally, an analysis of the relationships between environmental factors and snack intake showed that adolescents consumed significantly more unhealthy snacks when performing other activities while eating. In conclusion, the findings from this study suggest that families’ racial heritages are related to their parenting practices, BMI percentiles, and their adolescents’ food consumption and eating behaviors. The results of this study can be used to develop and improve adolescent nutrition education and interventions with consideration of their racial/ethnic backgrounds.

## 1. Introduction

Childhood obesity is a serious issue in the United States [1]. Based on the 2017–2018 data from the Centers for Disease Control and Prevention, the prevalence of obesity was 19.3% for children and adolescents aged 2–19 years, with a high prevalence among adolescents aged 12–19 years (with 22.2%). Importantly, the prevalence of childhood obesity is significantly higher among racial minority children in the United States, as compared to White children [2,3]. The data show that obesity prevalence among Hispanic children, non-Hispanic Black children, non-Hispanic White children, and non-Hispanic Asian children was approximately 26%, 24%, 16%, and 9%, respectively [1].

Parenting practices can contribute to childhood obesity, as parenting practices and the eating behaviors of adolescents are inextricably linked [4]. They form the foundation for long-term eating behaviors, which, in turn, are powerful predictors of long-term health outcomes [5]. Parents, as primary socialization agents, play a pivotal role in their adolescents’ healthy eating behaviors [6,7]. Therefore, small changes in parenting practices can influence adolescents’ healthy eating behaviors.

Several studies have examined the associations between various parenting practices and the consumption of different food categories. For example, Watts et al. (2017) revealed that availability, accessibility, modeling, and encouragement are independently associated with higher consumption of fruits and vegetables [8]. Similarly, another study reported a positive relationship between parents’ healthy modeling and children’s healthy eating index (HEI) scores [9]. Additionally, researchers have found that establishing rules and restrictive guidance for children are effective parenting practices for preventing the consumption of unhealthy foods [7]. Among the different parenting styles, authoritarianism has been identified as leading to a higher BMI percentile in adolescents [10].

Eating habits are impacted by racial differences. For instance, the frequency of fruit and vegetable intake is higher amongst the Hispanic population than non-Hispanic Black and non-Hispanic White [11,12]. Interestingly, acculturation among the immigrated Hispanic population in the US leads to a decrease in the consumption of fruits and vegetables [13]. A study that compared racial differences and eating habits amongst preschoolers reported a higher sugar-sweetened beverage and fast food consumption, and a lower intake of skim or 1% milk among Hispanic and black children than non-Hispanic White [14]. There is a gap in the literature that examines eating habit differences of different races/ethnicities specifically among adolescence.

Social cognitive theory (SCT) helps explain the theoretical framework in this study. This theory describes how healthy behaviors are influenced by individual experiences, environmental factors, and the actions of others [15]. Thus, SCT can explain how healthy eating behaviors among adolescents are influenced by racial differences and/or parenting practices/styles. Moreover, SCT suggests that self-efficacy is the greatest determinant of nutrition behaviors [15,16]. Self-efficacy constructs can be impacted by parenting practices/styles. Therefore, SCT can identify the most influential parenting practices/styles to promote healthy eating behaviors among adolescents. Parenting practices may also differ among various racial groups, and parents potentially adopt various parenting practices based on their racial backgrounds, although this has not been adequately studied [17,18]. Therefore, it is important to consider race/ethnicity while examining the effect of parenting practices on the eating behaviors of adolescents. However, no study has yet examined the relationship between different parenting practices and the types of foods consumed by adolescents while considering the racial background of the families. In this study, our objective was to identify the parenting practices adopted by members of different racial groups that led to healthy weights and the establishment of healthy eating behaviors among adolescents. We also explored the influences of different environmental factors on adolescents’ eating behaviors. By identifying parenting practices that can positively contribute to establishing healthy eating habits in adolescents, we will be better equipped to encourage parents to adopt these practices.

## 2. Methodology

### 2.1. Participants

Our study used interviews from parent–adolescent dyads as part of a multistate project that identified parenting practices and their influence on eating behavior. Fifty-one early adolescents aged 10–13 years old and their parents from 10 different U.S. states (i.e., Arizona, Utah, Georgia, Hawaii, Kansas, Minnesota, Ohio, Oregon, Indiana, and Connecticut) and the District of Columbia were interviewed. The inclusion criteria for parents were the ability to read and speak English, as well as living with and providing food for their adolescents.

### 2.2. Procedure

The protocol of the current study was approved by the institutional review boards (IRBs) of all participating universities. The parents filled out informed consent and parental permission forms. The adolescents completed a separate child assent. The participants met with the research team twice. In the first session, the study protocol was explained, the consent form was completed, and instructions were provided to adolescents for collecting food photos. A diary of each food item eaten during the day was recorded by adolescents taking food photos. The second session was an arranged interview based on the captured food photos, during which the interviewers asked the adolescents to describe each food item in each photo regarding the type, amount consumed, and the environmental context around the eating occasions (EOs). The interviews were conducted face-to-face, and the parents and adolescents were interviewed separately. All interviews were audio-recorded. The interviews for the parents lasted 60 min, while those for the adolescents were 30 min. In addition, a questionnaire for collecting demographic information was answered by parents. Parent and child height and weight and waist circumference were measured twice. Trained research assistants measured the parents’ and adolescents’ height, weight, and waist circumference twice. The parents’ body mass index (BMI) and the adolescents’ BMI-for-age percentile were calculated based on a standard protocol [18].

### 2.3. Parents’ Surveys and Interviews

The parents answered a demographic survey for their adolescents, themselves, and their households. Demographic questions included sex, age, household income, household food security, household acculturation, employment status, parents’ education level, and participation in any federal food assistance programs. Responses to the survey questions were reported using a 5-point Likert scale ranging from “strongly disagree” to “strongly agree” or “never” to “always” [19]. The parenting practices examined included reasoning, monitoring, modeling, and copying; authoritative, rule-setting, authoritarian, and neglectful were considered parenting styles. Scores for each parenting practice and style were assigned to each parent. Four food parenting practices were identified based on the parents’ interviews. The details of the interview procedure were described in the study by Gunther et al. (2019) [20]. The parenting style survey questions were adapted from the Comprehensive General Parenting Questionnaire (CGPQ) [21].

### 2.4. Adolescents’ Interviews

The adolescents’ consumed food items were categorized as either dairy, fruits and vegetables, or unhealthy snacks (i.e., sweets, salty snacks, and nondairy, sugar-sweetened beverages), and were retrieved from the adolescents’ interviews and identified through the food photos. Each food category’s details are listed in Table 1. Environmental variables, such as where adolescents ate (i.e., home, school, car/bus, or other) and their activities while eating (i.e., just eating vs. performing other actions) were considered in this study.

### 2.5. Data Analysis

An exploratory factor analysis with a varimax rotation was used to identify parenting practices and styles (*n* = 51) [21,22]. A Spearman’s rank correlation was used to test the association between the BMI and BMI percentiles of both the parent and adolescent in a dyad and parenting practices. Poisson regression models were used to examine the relationship between parenting practices and adolescent food consumption. Separate models were performed for each food group. All models controlled for the parents’ race/ethnicity. Generalized estimating equation models were used to account for the different number of EOs per adolescent. Separate models were performed for each food category (i.e., dairy, fruits and vegetables, and unhealthy snacks). For each food category, we calculated the total number of items consumed at each EO. The covariates considered were parent race/ethnicity, those parenting practice scores found to be related to consumption in the univariate analyses, adolescent BMI, household income, and parent education. Adolescent BMI, household income, and parent education were not related to consumption and were dropped from the final models. For fruit and vegetable consumption, monitoring and modeling were included as covariates; for unhealthy snack consumption, authoritative, reasoning, and rule-setting were included; no parenting practices or covariates were related to dairy consumption. The statistical analysis was performed using SAS 9.4 (SAS Institute, Cary, NC, USA), and a *p*-value of less than 0.05 indicated significance.

## 3. Results

### 3.1. Parenting Practices

Two exploratory factor analyses were run for sets of 35 and 33 items. Additional details of these analyses were reported by Monroe-Lord et al. (2021) [18]. The details of the items are listed in Table 2.

The exploratory factor analysis produced eight factors, with 3 out of the 68 items excluded from the final factors, as they did not load on any of the factors. Four factors addressed food-related practices and four factors addressed general parenting styles. The parents received a score on all eight parenting practices and styles. The factor loadings and variance for each factor are shown in Table 3.

### 3.2. Demographic Analysis

The sample’s demographic characteristics are presented in Table 4. The majority of the study population was female (59% of the adolescents and 88% of the parents). Seventy percent of the participants had an annual household income of less than USD 45,000. More than 75% of the parents had more than a high school education, and 42% were married.

### 3.3. Parenting Practices and Styles and Race/Ethnicity

The relationships between the race/ethnicity of the parents and their parenting practices and style scores are shown in Table 5. Overall, we found differences by race/ethnicity in three parenting practices and styles: Modeling (*p* = 0.029), authoritative (*p* = 0.018), and authoritarian (*p* = 0.009). In the pairwise comparisons, African American parents had higher mean scores on modeling than Hispanic (*p* = 0.03) and non-Hispanic White (*p* = 0.02) parents. In contrast, non-Hispanic White parents had lower scores on authoritative and authoritarian styles than both African American (*p* = 0.02 and *p* = 0.01, respectively) and Hispanic (*p* = 0.03 for both) parents.

### 3.4. Parenting Practices and Styles and Adolescent BMI Percentiles

Table 6 shows the relationship between parenting practices and styles and adolescent BMI percentiles by race/ethnicity. We found no significant relationships between BMI percentiles and parenting practices and styles, though two neared significance. Among non-Hispanic Whites, a higher monitoring score was related to a lower BMI in adolescents.

### 3.5. Adolescents’ Food Consumption by Parenting Practice and Style and Race/Ethnicity

No differences were observed in the number of fruits and vegetables or dairy items consumed based on race/ethnicity. In contrast, there was a significant difference based on race/ethnicity in terms of the number of unhealthy snacks consumed (*p* = 0.006). Non-Hispanic White adolescents consumed more unhealthy snacks than both Hispanic and African American adolescents in terms of the mean number of items consumed per EO. On average, African American and Hispanic adolescents consumed approximately half the number of unhealthy snacks (mean (SE): 0.36 (0.07) and 0.36 (0.06), respectively), as compared to non-Hispanic White adolescents (mean (SE): 0.66 (0.09)).

Table 7 shows the mean number of items consumed per EO by food group. There were no significant differences in dairy consumption related to parenting practices and styles. In contrast, three parenting practices, namely, modeling (*p* < 0.001), reasoning (*p* = 0.008), and monitoring (*p* < 0.001), were significantly related to the number of fruits and vegetables consumed. Higher scores for modeling, reasoning, and monitoring were predictive of more fruit and vegetable items consumed per EO. Similarly, reasoning (*p* = 0.024) and rule-setting (*p* = 0.039) were significantly related to the number of unhealthy snacks consumed per EO. Higher scores for the reasoning and authoritative styles were predictive of a lower number of unhealthy snack items consumed per EO. In contrast, higher scores for rule-setting were predictive of the consumption of fewer unhealthy snack items per EO.

Finally, two models were used to examine the relationships between the consumption of fruits and vegetables versus unhealthy snacks, and parenting practice and style versus parent race/ethnicity. Both modeling (*p* = 0.001) and monitoring (*p* = 0.008) were significant independent predictors of fruit and vegetable consumption, whereas parent race/ethnicity was not (*p* > 0.05). In these models, for predicting unhealthy food consumption, reasoning (*p* = 0.02) and rule-setting (*p* = 0.04) were significant independent predictors of unhealthy snack consumption. In the pairwise comparisons, African American adolescents consumed significantly fewer unhealthy snacks than non-Hispanic White adolescents (*p* = 0.03).

In the next step, multivariate Poisson regression models were used and Table 8 shows the detailed results of these models. Parent race/ethnicity was not significant in either model. In Model 1, both modeling and monitoring were associated with fruit and vegetable consumption. Higher monitoring and modeling scores were associated with higher fruit/vegetable consumption.

Table 9 shows the parent race/ethnicity and the modeling scores at the observed values, the monitoring score was 1 for all observations, and the average predicted number of fruits and vegetables consumed was 1.86. In contrast, at a monitoring score of 5, the average predicted number of fruits/vegetables consumed was 6.57. Similarly, with the parent race/ethnicity and monitoring score at the observed values, the average predicted number of fruits/vegetables consumed at modeling score 5 was 6.62 compared to 1.91 at modeling score 1. In Model 2, reasoning and rule-setting were significantly associated with unhealthy snack consumption. Higher reasoning and authoritative scores were associated with fewer snack items consumed. In contrast, higher rule-setting scores were associated with a higher predicted average number of unhealthy snack items consumed.

### 3.6. Environmental Factors, Family Race/Ethnicity, and Adolescents’ Sex

Overall, 76% of all EOs occurred at home, and 10% occurred at school. We found significant differences based on race/ethnicity in terms of the location of eating (*p* = 0.005). As compared to Hispanic and non-Hispanic White adolescents, African American adolescents had fewer EOs at home (61% vs. 82% for both) and more EOs at school (22% vs. 5% and 7%, respectively). Eating location also varied by sex (*p* = 0.001). Male adolescents had a larger proportion of EOs at home than female adolescents (88% vs. 67%), whereas female adolescents had 14% of EOs at school, as compared to 5% of male adolescents.

A difference was also identified based on race/ethnicity and sex in terms of activities while eating (*p* = 0.048). African American adolescents spent 30% of their eating opportunities not engaged in any other activity, as compared to 43% and 48% of non-Hispanic White and Hispanic adolescents, respectively. Male adolescents reported that half of their EOs were spent eating and not engaged in other activities, as compared to female adolescents, who reported that 34% of their EOs were spent just eating (*p* = 0.01).

## 4. Discussion

The relationship between an adolescent–parent dyad’s racial/ethnic background and parenting practice and style has not been sufficiently studied in the literature. The objective of this study was to examine this relationship, and we discovered that the modeling practice, as well as authoritative and authoritarian parenting styles, were significantly correlated with a family’s race/ethnicity. African American parents adopted these three parenting practices and styles more often than parents of other races/ethnicities. Our findings are consistent with a previously reported study showing that authoritarian methods were common among African American parents [23,24].

We also evaluated the relationships between parenting practices and styles and the weight of adolescents from different races/ethnicities. The results indicated that African American families that employed more authoritarian approaches had adolescents with higher BMI percentiles. This result shows an association between authoritarian parenting styles, characterized as rigid and strict, and negative health outcomes among African American adolescents. This finding agrees with another study that found authoritarian parenting to be associated with a higher BMI in adolescents [25]. In addition, such adolescents are more likely to experience obesity in adulthood due to the parenting practices applied during adolescence [10]. Our results also showed that non-Hispanic White families that employed more monitoring approaches had adolescents with lower BMI percentiles. This finding contrasts that of another study concerning Hispanic families, showing that Hispanic children who received a high monitoring score had a higher obesogenic dietary intake [10].

We also investigated the relationship between parenting practices and the eating behaviors of adolescents. The results revealed that the consumption of unhealthy snacks among adolescents decreased with the use of the reasoning parenting practice and the authoritative parenting style. Authoritative parenting is described as warm, protective, and supportive, where parents attempt to encourage their children to develop healthy eating habits [26]. Two systematic review articles previously revealed that children raised with authoritative parenting styles tend to eat healthier foods and have lower obesity rates [27,28]. Conversely, rule-setting was found to lead to an increase in the consumption of unhealthy snacks among adolescents. However, the authors of another study found that rule-setting may decrease unhealthy food consumption [7]. Notably, in their study, rule-setting was considered to be restrictive guidance, whereas, in our study, rule-setting was defined as determining expectations [7]. In addition, we found that modeling, reasoning, and monitoring parenting practices were positively correlated with adolescents’ fruit and vegetable consumption. This finding is similar to the results of a previous study that showed that children whose parents role-model fruit and vegetable intake consume more daily fruits and vegetables than others [7,9]. In addition, in agreement with our findings, studies have consistently found a positive association between reasoning practices and the consumption of healthy foods, such as fruits and vegetables [24,29,30]. Considering that the reported rate of fruit and vegetable consumption was lower than the recommended level for adolescents, it is important for parents to pay attention to their parenting practices and styles in order to encourage their adolescent children to consume more fruits and vegetables [31].

Among three food items, unhealthy snack consumption was about two times higher amongst non-Hispanic White adolescents in comparison with African American and Hispanic adolescents in the univariate results. However, the finding of multivariate regression, while controlling for possible cofounders, revealed that parent race/ethnicity was not related to the consumption of both fruits and vegetables and unhealthy snacks for all racial groups. Higher monitoring and modeling of parenting practices resulted in greater consumption of fruits and vegetables among all racial groups. This study agrees with the findings of the previous study that showed monitoring parenting practice was positively related to fruit and vegetable consumption among African American adolescents [18]. Furthermore, higher reasoning and authoritative parenting practices decreased the consumption of unhealthy snacks among all racial groups. Rule-setting increased the consumption of unhealthy snacks among all racial groups. As adolescence is the age of autonomy and peer influence, the rule-setting parenting style can play a negative role in adopting healthy eating behaviors.

Identifying environmental factors is also important, as they can play a key role in supporting healthy eating habits among adolescents. Many small changes in the surrounding environment can influence individual eating behaviors. The effects of environmental variables, such as engaging in other activities while eating and eating location (e.g., home, school, or car/bus), were investigated in the current study. Interestingly, unhealthy snacks were consumed significantly more while performing other activities during EOs. A previous study reported a similar finding, revealing a positive association between the consumption of pizza, fried foods, snacks, and sweets while watching television, whereas the consumption of fruits and vegetables showed a negative association with watching television [32]. Therefore, it is critical for parents to pay attention to the food consumption of their adolescents while they perform other activities. Furthermore, a previous study indicated a difference in the frequency of watching television across different racial groups—among the different groups, African American children watched television significantly more at mealtime, as compared to all other racial groups [33]. Finally, the African American adolescents in the current study were found to have a higher percentage of EOs at school than other races/ethnicities.

## 5. Conclusions

In this study, we focused on family racial/ethnic backgrounds, their relationship with parenting practices and styles, and their impact on the eating behaviors of adolescents. The results of this study indicate that the modeling practice, as well as authoritative and authoritarian parenting styles, are more common among African American families. The monitoring practice corresponded to a lower risk of obesity among African American and non-Hispanic White adolescents. In addition, reasoning, monitoring, and modeling practices contributed to greater fruit-and-vegetable consumption. The rule-setting practice increased unhealthy snack consumption. Finally, unhealthy snacks were consumed significantly more by adolescents when performing activities while eating, as compared to just eating.

The main limitation of this study was the small sample size for each racial/ethnic category and the cross-sectional nature of this study. The results of this study may be used to improve existing nutrition education interventions and to develop new interventions for parents while considering racial and cultural differences. Future research should be longitudinal, with a larger sample size, focusing on various racial/ethnic groups in the United States to examine the influence of cultural factors on the eating behaviors and health conditions of adolescents.

## Figures and Tables

**Table 1:** Consumed food categories by adolescents.

**Dairy Category**	**Food Items**
Dairy	Cheese (or foods made with cheese, including macaroni and cheese, alfredo, taco, pizza, sandwich, bean and cheese burrito, egg and cheese bagel, etc.); yogurt, including those with additional “mix-ins” (e.g., M&M YoCrunch); white milk
Dairy SSBs ^1^	Chocolate milk; drink mixes (e.g., Nesquik vanilla or strawberry powder mix); ice cream milkshake
**Fruits and Vegetables Category**	**Food items**
Total fruits	Oranges (including mandarins); grapes; pears; pomegranates; mangoes; Gogo squeeze-pouch applesauce; bananas; cantaloupes; apples; pineapples; strawberries; juices (e.g., orange, cranberry, apple, and Capri-Sun 100% juice pouches)
Whole fruits	Oranges (including mandarins); grapes; pomegranates; mangoes; apples; squeeze-pouch applesauce (e.g., Gogo SqueeZ); bananas; cantaloupes; pineapples; pears; strawberries
Vegetables	Tomato/marinara sauce (on spaghetti); pizza sauce on pizza; carrots; tomatoes; broccoli; beans/legumes, including bean dips; lettuce; corn; sub-type sandwich w/vegetables; potatoes (sweet potatoes, potato salad, mashed, etc.); cabbage; okra; soup; vegetable juices (e.g., V8 tomato juice)
**Unhealthy Food Category**	**Food items**
Nondairy SSBs	Punch-type drinks and mixes (e.g., Hi-C orange drink, POG passion fruit juice, and Aloe Vera King juice); sugar-sweetened tea; sports drinks (Gatorade, Powerade, etc.); sugar sodas (Coke, Pepsi, etc.)
Sweet	Ice cream; sweetened grains (e.g., Pop-Tart breakfast pastries, funnel cake, chocolate-covered pretzels, cornbread, muffins, doughnuts, and granola bars); pies; marshmallows; cookies/cookie bars; brownies; cakes; trail-mix bars; fruit-flavored snacks (i.e., Fruit by the Foot); chocolate syrup; chocolate/jelly candies (e.g., M&Ms, Mentos, Lifesavers, Starburst, Twix, Tootsie rolls, lollipops, and Push Pop)
Salty snacks	Crackers (e.g., Cheez-It and round butter crackers); chips (e.g., Pringles, Doritos, flavored chips, tortilla chips, Hot Fries, Hot Cheetos, Funyuns, and Lay’s Stax); popcorn; pizza snacks/rolls/bagels; pretzels; “tater tots”

^1^ SSBs, sugar-sweetened beverages. Retrieved from [17].

**Table 2:** The items of the parental survey to identify parenting practices as obtained from the CGPQ and interviews.

**Parenting Practices**	**Questions**
Monitoring	1. How much do you keep track of the snack foods (potato chips, Doritos, cheese puffs) that your child eats? 2. How much do you keep track of the sugary drinks (soda/pop, Kool-Aid) that your child drinks? 3. How much do you keep track of the high-fat foods (fried foods, French fries) that your child eats? 4. How much do you keep track of the sweets (candy, ice cream, cake pastries) that your child eats? 5. How much do you keep track of the fruits and vegetables your child eats? 6. I like to be sure that my child does not eat too many high-fat foods. 7. I like to be sure that my child does not eat too many sweets (candy, ice cream, cake, or pastries). 8. I intentionally keep some foods out of my child’s reach.
Reasoning	1. How often do you say something positive about the food that your child is eating? 2. I explain my food choices verbally to my child (e.g., “I think I’m going to have some fruit, as I like it and it’s good for me”). 3. How often do you compliment your child for eating food (e.g., “What a good boy! You’re eating your vegetables”)? 4. How often do you encourage your child to try to eat healthy foods such as vegetables? 5. I try to talk more often about foods I would like my child to eat. 6. How often do you tell your child that healthy food tastes good? 7. I make comments about my eating behaviors/food choices when I am with my child (e.g., “I’ll be healthy and have vegetables”). 8. I verbally encourage my child to copy my eating behaviors. 9. How often do you reason with your child to get him/her to eat (e.g., “Milk is good for your health because it will make you strong”)? 10. I tend to talk more often about foods I would like my child to eat. 11. How often do you tell your child how tasty a new food is? 12. I try to influence my child’s food preferences by verbally stating my own (e.g., “I love carrots, they’re one of my favorites”).
Modeling	1. How much do you keep track of foods labeled as whole grain that your child eats? 2. My child is more likely to try new foods he or she has seen me eating. 3. When I show my child I enjoy fruits and vegetables, he or she tries them. 4. My child asks to try foods from my plate that he or she sees me eating. 5. My child has picked up eating behaviors from me which I have not intentionally encouraged him or her to copy (e.g., putting ketchup on most foods, or eating vegetables first). 6. My child is more likely to try or eat new foods if I eat the new foods with him or her. 7. How much do you keep track of the milk or foods with calcium, like cheese and yogurt, your child consumes?
Copying	1. My child has picked up eating behaviors from me which I had tried to hide from him or her (e.g., avoiding certain foods). 2. The eating behaviors of other family members influence what my child eats. 3. If I point out certain eating behaviors or foods I like or don’t like, my child is more likely to copy them. 4. My child has copied eating habits from me which I did not realize I had (e.g., salting my food before I taste it).
**Parenting Styles**	
Authoritative	1. I attend as many of my children’s events and activities as possible. 2. I know exactly when things are not going very well for my child. 3. My child and I have warm affectionate moments together. 4. I know exactly when my child has difficulty with something. 5. I easily find a way to make time for my child. 6. Every free minute I have I spend with my child. 7. I always help my child with everything he/she does. 8. I find it interesting and educational to be with my child for long periods. 9. When my child is sad, I know what is going on with him or her. 10. I feel good about the relationship I have with my child. 11. I find time to talk with my child. 12. I spend a lot of time with my child.
Rule-Setting/Expectations	1. I expect my child to follow our family rules. 2. I make sure that my child understands what I expect of him or her. 3. I have clear expectations for how my child should behave. 4. I teach my child to follow rules. 5. I have clear expectations for how my child should behave.
Authoritarian	1. When my child has lost something, I stop what I am doing to find it before he/she gets too upset. 2. I do not let my child get involved in activities or tasks where he/she may potentially fail. 3. I make my child feel guilty when he or she does not meet my expectations. 4. When my child hurts my feelings, I stop talking to him/her until he or she pleases me again. 5. I do not allow my child to question my decisions. 6. When I ask my child to do something, I expect him/her to do it immediately without any questions. 7. I let my child know that I am the boss in our house. 8. I do not allow my child to get angry with me. 9. I make sure my child is aware of how much I sacrifice for him or her. 10. I teach my child to stay in control of his or her feelings at all times.
Neglecting	1. When my child does something that is not allowed, I do not talk to him or her until he or she says he or she is sorry. 2. I threaten discipline more often than I actually give it. 3. There are times I just do not have energy to make my child behave as he or she should. 4. I have a hard time consistently enforcing rules with my child. 5. When I discipline my child, I sometimes end the punishment early. 6. I do not always follow through when I threaten to discipline my child. 7. I am less friendly with my child if he or she does not see things my way.

Retrieved from [18]. 5-point Likert scale ranging from “strongly disagree” to “strongly agree” or “never” to “always” was used to respond to the questions.

**Table 3:** Factor analysis for parenting practices and styles.

	*n*	Factor Loadings (Min–Max)	% of Variance Explained	Mean (SD)	Median (IQR)
Parenting practice					
Reasoning	8	0.42–0.83	5.70%	3.59 (1.01)	4 (3–4.4)
Monitoring	12	0.45–0.85	5.30%	3.66 (1.05)	3.7 (3.1–4.5)
Modeling	7	0.44–0.82	4.10%	3.72 (1.22)	4 (3.6–4.4)
Copying	4	0.60–0.88	3.30%	3.59 (1.44)	4.1 (2–4.8)
Parenting style					
Authoritative	12	0.49–0.83	7.40%	4.41 (0.95)	4.8 (4.4–4.9)
Rule-setting	5	0.78–0.87	5.10%	4.56 (1.07)	5 (4.4–5)
Authoritarian	10	0.43–0.84	4.90%	3.36 (0.91)	3.2 (2.7–3.8)
Neglecting	7	0.47–0.67	3.70%	2.78 (1.09)	2.6 (1.7–3.3)

**Table 4:** Demographic data.

Characteristics	*n*	%
**Adolescent age (years)**		
10	24	47.06
11	11	21.57
12	7	13.73
13	9	17.65
**Adolescent sex**		
Male	21	41.18
Female	30	58.82
**Adolescent race/ethnicity**		
African American	12	23.53
Hispanic	20	39.22
Non-Hispanic White	12	23.53
Other	7	13.73
**Parent age (years)**		
26–34	20	39.22
35–54	29	56.86
55 or over	2	3.92
**Parent sex**		
Male	6	11.76
Female	45	88.24
**Parent education**		
Below high school	2	3.92
Diploma or GED	10	19.61
Some college or technical school	27	52.94
Four-year college and above	12	23.53
**Parent race/ethnicity**		
African American	12	23.53
Hispanic	15	29.41
Non-Hispanic White	16	31.37
Other	8	15.69
**Household income (USD)**		
Below 24,999	16	31.37
25,000–44,999	20	39.22
45,000–64,999	6	11.76
65,000–84,999	4	7.84
Prefer not to answer	5	9.8
**Marital status**		
Single	17	34
Married	21	42
Separated	2	4
Divorced	8	16
Widowed	1	2
Never married	1	2

**Table 5:** Comparison of parenting practice and style scores by race/ethnicity.

Parenting Practice	*p*-Value	African American Mean Score (SD)	Hispanic Mean Score (SD)	Non-Hispanic White Mean Score (SD)
Reasoning	0.232	22.95 (36.80)	17.73 (39.10)	25.28 (39.65)
Monitoring	0.093	25.12 (36.88)	25.26 (39.19)	16.59 (39.74)
Modeling	0.029	30.12 (36.77)	18.53 (39.07)	19.15 (39.63)
Copying	0.358	25.33 (36.74)	22.86 (39.03)	18.68 (39.59)
**Parenting style**				
Authoritative	0.018	25.16 (32.77)	22.14 (34.06)	12.92 (33.47)
Rule-setting	0.086	25.54 (31.07)	16.42 (32.29)	18.73 (31.73)
Authoritarian	0.009	25.41 (32.81)	22.46 (34.10)	12.34 (33.51)
Neglecting	0.743	18.75 (32.78)	19.25 (34.06)	21.96 (33.48)

**Table 6:** Correlation between parenting practices and adolescent BMI percentiles by race/ethnicity.

	African American Adolescents’ BMI		Hispanic Adolescents’ BMI		White/Non-Hispanic Adolescents’ BMI	
**Parenting Practice**	**Correlation**	***p*-Value**	**Correlation**	***p*-Value**	**Correlation**	***p*-Value**
Reasoning	−0.18	0.600	0.08	0.769	−0.28	0.283
Monitoring	−0.18	0.608	0.29	0.286	−0.46	0.074
Modeling	0.19	0.572	0.09	0.739	−0.10	0.714
Copying	0.32	0.336	−0.01	0.979	−0.42	0.107
**Parenting style**						
Authoritative	0.10	0.766	0.11	0.705	0.22	0.464
Rule-setting	0.01	0.968	−0.43	0.124	0.03	0.919
Authoritarian	0.53	0.096	0.28	0.330	0.00	1.00
Neglecting	0.10	0.768	1.16	0.588	0.31	0.297

**Table 7:** Mean number of food items consumed per EO by parenting practice and style.

	Fruits and Vegetables		Dairy		Unhealthy Snacks	
	Mean (SE)	*p*-Value	Mean (SE)	*p*-Value	Mean (SE)	*p*-Value
**Ethnicity**		0.336		0.231		0.006
African American	0.76 (0.10)		0.33 (0.07)		0.36 (0.07)	
Hispanic	0.83 (0.10)		0.19 (0.05)		0.36 (0.06)	
White/non-Hispanic	0.64 (0.08)		0.22 (0.05)		0.66 (0.09)	
**Parenting practice**						
Reasoning	0.25 (0.10)	0.008	0.20 (0.17)	0.225	−0.26 (0.11)	0.024
Monitoring	0.37 (0.11)	<0.001	0.26 (0.18)	0.149	−0.19 (0.12)	0.137
Modeling	0.39 (0.11)	<0.001	0.01 (0.14)	0.914	−0.16 (0.10)	0.134
Copying	0.08 (0.08)	0.079	0.09 (0.13)	0.471	−0.09 (0.11)	0.421
**Parenting style**						
Authoritative	0.05 (0.13)	0.692	0.08 (0.21)	0.681	−0.07 (0.15)	0.060
Rule-setting	−0.04 (0.09)	0.150	−0.02 (0.15)	0.880	0.08 (0.13)	0.039
Authoritarian	−0.15 (0.11)	0.179	−0.0007 (0.19)	0.997	0.02 (0.14)	0.866
Neglecting	0.10 (0.09)	0.254	−0.06 (0.15)	0.697	−0.04 (0.11)	0.726

**Table 8:** Multivariate regression models for fruits and vegetables and unhealthy snack consumption.

**Model 1**	**Fruit and Vegetable Consumption**		
		**Estimate (SE)**	***p*-Value**
	**Ethnicity**		0.58
	African American	−0.21 (0.21)	0.31
	Hispanic	0.15 (0.21)	0.49
	White/non-Hispanic	Ref	
	**Parenting practice**		
	Monitoring	0.37 (0.12)	<0.01
	Modeling	0.30 (0.11)	<0.01
**Model 2**	**Unhealthy Snack Consumption**		
		**Estimate (SE)**	***p*-Value**
	**Ethnicity**		0.08
	African American	−0.52 (0.24)	0.03
	Hispanic	−0.39 (0.26	0.13
	White/non-Hispanic	Ref	
	**Parenting Practice/Style**		
	Reasoning	−0.29 (0.12)	0.01
	Authoritative	−0.41 (0.23)	0.07
	Rule-setting	0.37 (0.19)	0.04

**Table 9:** Race/ethnicity and parenting practices/styles and the average number of fruits and vegetables and consumption of unhealthy snacks.

	Fruits and Vegetables		Unhealthy Snacks		
Score ^1^	Monitoring ^2^	Modeling ^3^	Reasoning ^4^	Authoritative ^5^	Rule-Setting ^6^
1	1.86	1.91	5.97	10.81	0.82
2	2.55	2.6	4.46	7.15	1.19
3	3.5	3.55	3.34	4.47	1.73
4	4.79	4.85	2.49	3.14	2.51
5	6.57	6.62	1.86	2.08	3.64

^1^ Parenting practices/style scores. ^2^ Race/ethnicity and modeling held at observed scores. ^3^ Race/ethnicity and monitoring held at observed scores. ^4^ Race/ethnicity, authoritative, and rule-setting held at observed scores. ^5^ Race/ethnicity, reasoning, and rule-setting held at observed scores. ^6^ Race/ethnicity, reasoning, and authoritative held at the observed scores.

## Data Availability

The data used during the current study are available from the corresponding author upon request.
